# Highlights from the 8^th^ World Congress on Peritoneal Surface Malignancies

**DOI:** 10.7497/j.issn.2095-3941.2012.04.011

**Published:** 2012-12

**Authors:** Yan Li

**Affiliations:** Hubei Cancer Clinical Study Center & Hubei Key Laboratory of Tumor Biological Behaviors, Department of Oncology, Zhongnan Hospital, Wuhan University, Wuhan 430071, China

The 8th World Congress on Peritoneal Surface Malignancies was held in Berlin, Germany from October 31, 2012 to November 2, 2012, in which more than 571 delegates from 56 countries attended. This conference covered a variety of topics on peritoneal carcinomatosis (PC) from gastric, colorectal, and ovarian cancers, as well as pseudomyxoma peritonei, malignant peritoneal mesothelioma, and abdominal sarcoma. PC experts across the world presented the most up-to-date results of their basic and translational studies as well as clinical trials. In particular, the following research interests were highlighted.

Professor Paul H. Sugarbaker from the Washington Hospital Cancer Center, Washington DC, USA, systemically reviewed the developments in PC treatment for the past 30 years. He stressed that a combination of cytoreductive surgery (CRS) and hyperthermic intraperitoneal chemotherapy (HIPEC) has been generally accepted by oncologists and should be regarded as the standard treatment for PC from the following malignancies: *i*) pseudomyxoma peritonei; *ii*) appendiceal tumors with perforation that leads to the spread of tumor cell into the abdominal cavity; *iii*) malignant peritoneal mesothelioma with a peritoneal cancer index (PCI) of less than 20; *iv*) PC from colorectal cancer when no distant metastases or retroperitoneal lymph nodes are involved.

Professor Pompiliu Piso from Germany presented the most recent achievement in colorectal PC treatment. In 2013, the German Guideline for Colorectal Cancer will fully promote CRS+HIPEC as the standard therapy for PC from colorectal cancer. The new guideline states the following: *i*) For patients with isolated and limited PC but without extraabdominal metastases and low PCI (<20), CRS+HIPEC can be performed; *ii*) A pre-requisite is a complete macroscopic cytoreduction and a potential benefit for the individual patient in accordance with the interdisciplinary tumor board decision; *iii*) The treatment should be performed in specialized centers. If possible, patients should be included in studies. Moreover, a national network on colorectal PC has been established.

Professor Marcello Deraco from Milan, Italy, is also a pioneer in this field, with special achievements in pseudomyxoma peritonei and malignant peritoneal mesothelioma studies. In his recent translational study, he found that serum thioredoxin-1 levels are related to the progression of peritoneal mesothelioma and serum CA153 levels are associated with the residual tumor burden after surgery. Moreover, he developed pharmacogenetic microarray chips to guide the individualized treatment of PC from colorectal cancer, pseudomyxoma peritonei, and mesothelioma.

Olivier Gelhen, a researcher from France, presented his multicenter studies in France on PC from colorectal and gastric cancers. He is currently leading a randomized multicentric phase III trial, called GASTRICHIP, to prevent the onset of PC from gastric cancer. This study is supported by a special grant from the NIH. Once completed, it will provide new insights in the prevention of PC from gastric cancer.

Professor Vic Verwaal from the Netherlands reported his experience in colorectal PC treatment. More than 600 patients with colorectal PC have been treated with CRS+HIPEC. The most remarkable achievement includes 5- and 10-year survival rates that reached 50% and 25%, respectively. This study has provided one of the most convincing evidence that the new treatment strategy brings significant survival benefits to colorectal cancer patients with PC.

Professor Yutaka Yonemura from Japan presented his video demonstration on CRS+HIPEC for PC from gastric cancer. He also reported his bi-directional strategy for the reduction of the tumor burden of gastric cancer before CRS. The patient initially receives both systemic chemotherapy and intraperitoneal chemotherapy for 2 to 4 cycles. Once the tumor has been under adequate control, a complete CRS+HIPEC can improve a patient’s survival by >60%.

Professor Yan Li from China also reported his systemic studies on PC from gastric cancer. His group has focused on this topic for 10 years already. Based on four animal model studies used to confirm the safety and efficacy of CRS+HIPEC, phases I, II, and III of the clinical trials on PC from gastric cancer were successfully conducted. Results showed that PC patients with gastric cancer only have a medial overall survival of 6.5 months under the current conventional treatment. However, if the patients receive CRS+HIPEC, the medial survival can reach 11.0 months; for those with synchronous PC from gastric cancer, the survival can reach 12.0 months. Moreover, a long-term follow-up of the extension study confirmed the results.

Given that CRS+HIPEC has been increasingly used worldwide, a more standardized approach should be established. The delegates discussed international collaborations to set up regional training centers and technical transfer facilities.

This conference has academic and clinical significance to oncologists. From the Oncology Sciences perspectives, three forms of cancer metastases are found, namely, lymph node metastases, distant metastases, and peritoneal metastases. For lymph node metastasis, universally-adopted treatment guidelines and technical standards have been established in which surgery plays a major role. For distant metastases, such as liver metastasis from colorectal cancer, surgery is considered the treatment of choice for selected patients with limited liver metastasis and good function reserve. Compared with these two forms of cancer metastases, peritoneal metastasis is the least form of cancer therapy. Given this problem, the oncology community as a whole, together with the medical field in a larger sense, remains pessimistic because palliative approaches have not resulted in concrete benefits other than psychological treatment to patients and doctors.

This nihilistic approach is ill-conceived in theory and counter-productive to clinical oncology. Experiences and achievements for the past three decades have unequivocally proven that special features account for the development of PC, which can be considered in the development of more proactive therapeutic strategies. Among the current treatment approaches, CRS+HIPEC is the only method that has been proven to provide real survival benefits to patients. Therefore, well-designed clinical studies should be conducted under the CRS+HIPEC framework to improve the efficiency of the strategy, which is less time-consuming and more accessible to clinicians.

This meeting is of particular importance for China, where cancer has emerged as the major cause of deaths for the past decade. The incidence has been continuously increasing for several years. For the cancer spectrum in China, gastrointestinal and gynecological cancers are among the most frequent malignancies. These cancers are prone to developing PC once the tumor is beyond clinical stage III. Therefore, PC is a more acute clinical problem for Chinese oncologists. However, this problem has not been paid enough attention in China and the prevailing practice has not tackled it seriously. With the promising results of CRS+HIPEC, clinicians should exhibit a proactive attitude toward this problem to learn and practice the new treatment approach. In addition, new healthcare policies are needed to encourage more specialized cancer centers to implement such treatment. Well-designed and well-organized clinical studies are also urgently needed to make the CRS+HIPEC strategy more suitable to the clinical requirements of Chinese patients. Through concerted efforts from doctors, patients, and policymakers, China would develop more effective treatment approaches for PC.

